# A Region on Chromosome 7 Related to Differentiation of Rice (*Oryza sativa* L.) Between Lowland and Upland Ecotypes

**DOI:** 10.3389/fpls.2020.01135

**Published:** 2020-07-27

**Authors:** Md. Nashir Uddin, Yoshimichi Fukuta

**Affiliations:** ^1^ Department of Biochemistry and Microbiology, School of Health and Life Sciences, North South University, Dhaka, Bangladesh; ^2^ Tropical Agricultural Research Front, Japan International Research Center for Agricultural Science (JIRCAS), Ishigaki, Japan

**Keywords:** differentiation, upland, lowland, ecosystems, plant architecture, rice (*Oryza sativa* L.)

## Abstract

Due to global population expansion and climate change impacts, the development of a stable yielding variety that adapts well to unfavorable conditions for rice cultivation, can contribute to sustainable and stable production in rice (*Oryza sativa* L.). Understanding genetic differentiations to ecotypes for rice cultivations, such as upland, rainfed lowland, and irrigated lowland, is very important to develop the breeding materials for adapting to each environmental condition. The upland landrace variety basically has low tiller/panicle numbers and a large panicle, and the plant architecture is different from that of the lowland variety. The tiller and panicle numbers have been considered as one of the most difficult traits for genetic changes artificially in rice breeding. A low tiller recessive gene *ltn2* originated from a New Plant Type variety, IR 65600-87-2-23, harboring segments from an upland variety, Ketan Lumbu (Tropical Japonica Group), was found on chromosome 7, and the other QTLs for culm length, culm weight, panicle length, panicle weight, seed fertility, harvest index, and soil surface rooting were also detected in the same chromosome region. These low tiller genes and the other QTLs were estimated to play an important role in developing the architecture for upland rice. Some QTLs for root growth angle, *DRO3* and *qSFR7*, were also found in the same chromosome region from upland varieties categorized into the Tropical Japonica Group, and the QTLs may also be relevant to upland adaptation together with other traits. Previous studies using high throughput re-sequencing (whole genome variation data) of a large batch of rice accessions could identify the ecotype differentiated genomic regions (EDRs) and Ecotype differentiated genes (EDGs) such as *Os07g0449700*, a type response regulator, which is critical in upland adaptation in the same region of chromosome 7. Two selective loci, *E3735* and *E4208*, for upland and lowland differentiation, and their corresponding genes *Os07g0260000* and *Os07g0546500* were also detected on chromosome 7 by drought-responding EST-SSRs. These findings indicate that the region on chromosome 7 is highly possible to related to the plant shoot and root architecture in the upland rice variety that has an important role and differentiates between upland and lowland ecotypes.

## Introduction

Climate change is the main causal element or factor of biotic and abiotic stresses, which have negative effects on global food production including rice (*Oryza sativa* L.) (Raza et al., 2019). Recently, one study estimated a 15% decrease of rice yield in irrigated conditions of developing countries and a 12% increase in rice price as a result of climate change by 2050 (IFPRI, 2009). By contrast, current estimates showed that rice production had to increase by over 20% before 2030 to satisfy the demands of the world’s growing population (Purevdorj and Kubo, 2005; FAO RMM, 2018) and to avoid food crises.

The development of a stable yielding variety that adapts well to unfavorable conditions for rice cultivation, such as rainfed lowland and upland ecosystems, can contribute to sustainable rice production and ensure global supply. The respective characteristic traits of rice varieties have been modified under both natural and artificial selections, which lead to phenotypical adaptation to the respective environment and genetic differentiation at the same times between ecotypes (Lyu et al., 2014). Under the upland or rainfed lowland ecosystems, rice cultivation needs to reduce water use in rice production and increase the water use efficiency, and from an environmental perspective, emission of methane is substantially lower in this condition (Xia et al., 2014). Understanding the genetic differentiation among upland, rainfed lowland, and irrigated lowland rice is very important for rice breeding in unfavorable conditions. Especially, rice varieties between upland and irrigated lowland differ significantly in phenotypical and physiological traits. Particularly, the upland variety basically has low tiller/panicle numbers, tick and long culm, and a large panicle, and the plant architecture is different from that of lowland varieties.

Several studies have been conducted to fine the genetic factor(s) for differentiation of ecotypes between lowland and upland. Ishikawa et al. (1992) found that the allele of isozyme gene *Pgd-1* locating chromosome 11 (Wu et al., 1988) was related with the differentiation between Japanese upland varieties and Japanese lowland ones. Zheng et al. (2000) detected three QTLs, *qPRN7* for root penetration ability, *qRN7* for root numbers, and *qRPI* for root penetration index on chromosome 7 in a wax-petroleum layer system simulated to Asian upland soil. Thick root and high penetration ability are important for drought resistance in upland soil. Two deeper rooting QTL, *DRO3* by Uga et al. (2015) and *qRDR7* by Luo et al. (2015) which regulate root-growth-angle of upland rice were also detected on chromosome 7. Uddin et al. 2016a; 2016b) described *qPN7* for panicle number and a gene for low tiller number, *ltn2*, which constrict plant architecture in upland on chromosome 7. Monkada et al. (2001) and Lafitte et al. (2004) reported *qDTH7, qSF7* and *qGW7*, which extended the heading date and increased the spikelet fertility and per grain weight in upland. Xu et al. (2020) recently reported upland specific QTLs, *qHD7*, *qGY7*, and *qHI*, which plays important role in reproduction and yields in upland. Three candidate genes; *Os07g0449700* by Lyu et al. (2014), *Os07g0260000* and *Os07g0546500* by Xia et al. (2014), were also reported as ecotype differentiation genes on chromosome 7 which regulate the root system and plant height in upland rice varieties and help with drought tolerance or resistance. Thus, some chromosome regions were estimated to related with the genetic differentiation between lowland and upland ecosystems, and many QTLs genes for agronomic traits differentiating between ecotypes in lowland and upland have been detected (**Table 1**).

**Table 1:** List of genes and QTLs on chromosome 7 for ecotype differentiation.

QTL or gene detected	Physical position (Mb) of the closest marker*	Regulatins/Functions	Origin	Ecotype	Reference
*DRO3*	24.2	Root angle	Kinandang Patong	Upland	Uga et al. (2015)
*qSOR1*	25.6	Surface rooting	Gemdjah Beton	Lowland	Uga et al. (2012)
*qPN7^b,d^*	24.5	Panicle No.	IR65600-87-2-2-3	Upland	Uddin et al. (2016a)
*qTN7* and *ltn2*	25.1	Low tiller No.	IR65600-87-2-2-3	Upland	Uddin et al. (2016b)
*qSF7*	26.0	Spikelet fertility & Root depth	Azucena	Upland	Lafitte et al. (2004)
*qGW7*	26.0	Weight per Grain & Root depth			
*qSFR7*	23.6	Soil Surface rooting	IR65600-87-2-2-3	Upland	Tomita and Fukuta (2019)
*qHD7*	25.5	Heading date	B61144F-MR-6	Upland	Xu et al. (2020)
*qGY7*	25.5	Grain yield			
*qHI7*	25.5	Harvest index			
*qRDR7*	26.0	Deep rooting	IRAT 109	Upland	Luo et al. (2015)
*qBRT^a^*	19.0	Root thickness		Lowland	Li et al. (2011)
*qBRT^b^*	25.9	Root thickness		Lowland	Li et al. (2011)
*qRN^a^*	29.6	Root number		Upland	Li et al. (2005)
*qDTH7*	24.8	Heading date	*O. rufipogon (IRGC #105491)*	upland	Monkada et al. (2001)
*qPRN7*	17.8	Penetrated root numbers	Azecena	Upland	Zheng et al. (2000)
*qTRN7*	14.4	Total root numbers			
*qRPI*	28.4	Root penetration index			
*Os07g0449700*	16.1	Root and shoot development	Panel of upland varieties	Upland	Lyu et al. (2014)
*Os07g0260000 Os07g0546500*	9.1	Drought tolerance	Panel of upland varieties	Upland	Xia et al. (2014)

*Physical position of the closest markers were retrieved from the genome database in Gramene (http://www.gramene.org/) and Oryzabase (https://shigen.nig.ac.jp/rice/oryzabase/). DRO, deeper rooting; SOR, soil surface rooting; BRT, basal root thickness; RDR, ratio of deep rooting; PN, panicle number; TN, tiller number; HI, harvest index; GW, grain weight; SF, spikelet fertility; GW, grain weight; DTH, days to heading; DH, heading date; RP, root penetration; RN, root number; PRN, penetrated root number.

Interestingly, most of the research findings indicated that a region on chromosome 7 was related to the variations of unique traits between the two ecotypes, such as drought tolerance/avoidance and upland adaptation. This article review examines the involvement of chromosome 7 in the genetic differentiation of upland and lowland varieties, and then discusses how this information can be used in breeding a rice variety that adapts well to unfavorable conditions for sustainable production in rice.

## A Low Tiller Gene Contributing to Plant Architecture

Plant architecture with a low tiller and high-density grain is desirable in limited water conditions to maintain a proper plant density, to avoid episodic drought, and to keep the stable yield production based on the minimizing of reproductive tillers under the serious conditions, such as rainfed lowland and upland (Vergara et al., 1990; Farooq et al., 2011). The control for numbers of tiller and panicle may be the important breeding target for increasing the adaptability and seed production in the growing stages from productive to reproductive stages of rice against upland and serious environmental conditions.

Several artificial mutant genes for tiller numbers, *tdr2* (Hasegawa et al., 2005) and *rcn9* (Jiang et al., 2006) on chromosome 1, *OsTB1* on chromosome 3 (Takeda et al., 2003), *HTD1* on chromosome 4 (Zou et al., 2005), and *D3* (Ishikawa et al., 2005), *MOC1* (Li et al., 2003), and *rcn8* (Jiang et al., 2006) on chromosome 6, have been found in rice. These genes are associated with branching mechanisms of the tiller. As the natural variations, many QTLs for numbers of panicles and tillers have been reported. Lin et al. (1996) found them on chromosomes 2, 4, 5, and 6; Wu et al. (1998) were on chromosomes 1, 3, and 5; Yan et al. (1998) were on chromosomes 1, 2, 3, 4, 5, 6, 7, 8 and 12; Nagata et al. (2002) were on chromosome 1; Hittalmani et al. (2003) were on chromosomes 1, 3, 4, and 12; Miyamoto et al. (2004) were on chromosomes 2, 5, 6, and 8; Liu et al. (2008; 2010; 2012) were on chromosomes 1, 2, 3, 4, 6, 7, 8, 9, and 12, and Bian et al. (2013) were on chromosomes 1, 2, 4, 5, 6, 7, 8, and 10. A low tiller-number gene, *Ltn* was found, on chromosome 8 in a Japanese Japonica Group variety, Aikawa 1 (Fujita et al., 2010). Thus, several genes and many QTLs have been identified almost rice chromosomes except for chromosome 11, as the genetic factors for controlling the tiller and panicle numbers in rice. These QTLs for tiller or panicle numbers by Yan et al. (1998), Liu et al. (2008; 2010; 2012), and Bian et al. (2013) were detected in the similar region of long arm on chromosome 7.

**Figure 1:**
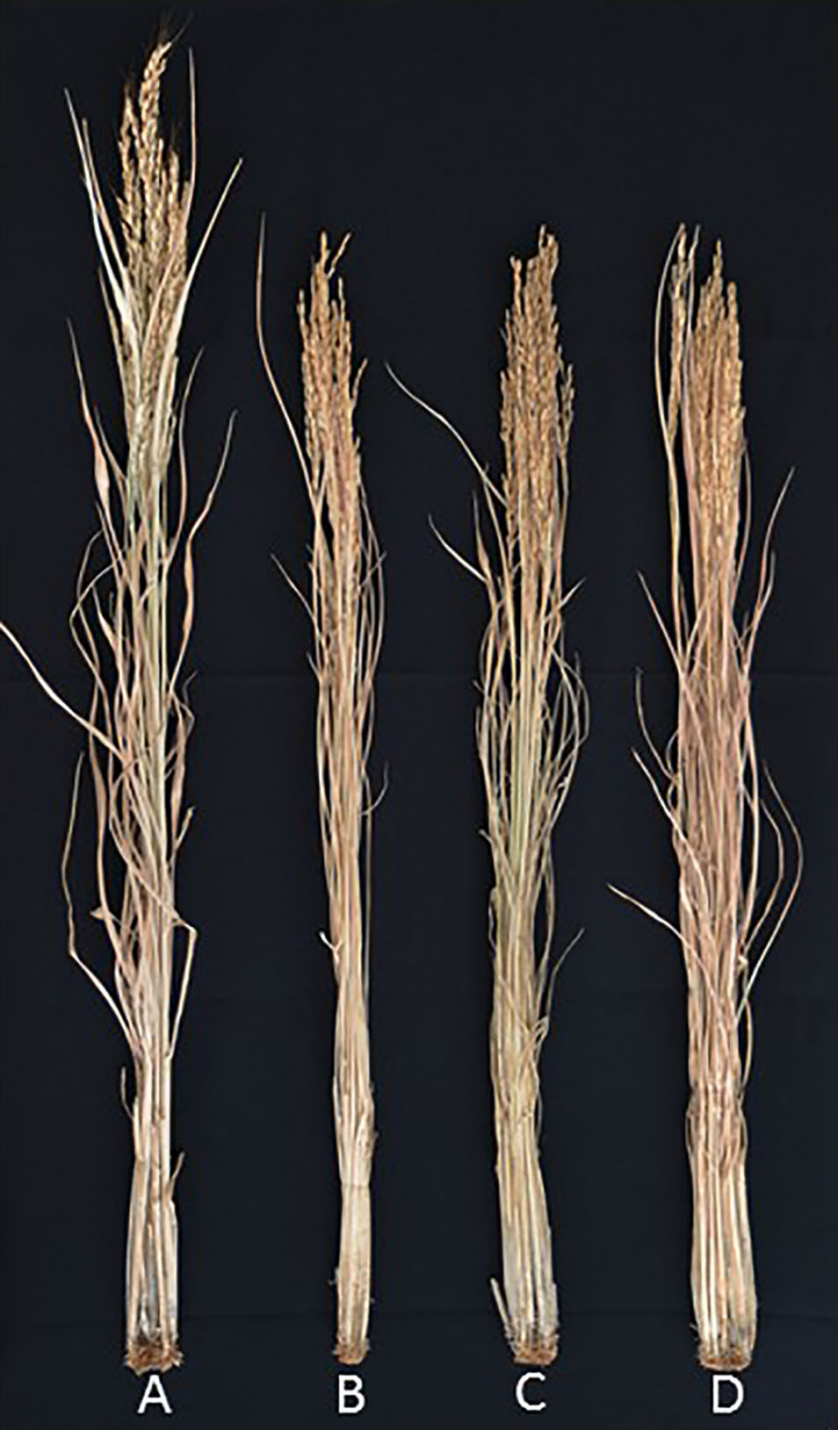
Phenotypic characteristics of an introgression line for *Itn2* originated from an IR 65600-87-2-2-3. Photo of rice plants at maturity stage. Rice was cultivated in lowland field at Tropical Agricultural Research Front, JIRCAS, Ishigaki, Okinawa, Japan, in 2015. **(A)** IT 6600-87-2-2-3, **(B)** YTH16, **(C)** YTH34, **(D)** IR 64.

**Figure 2:**
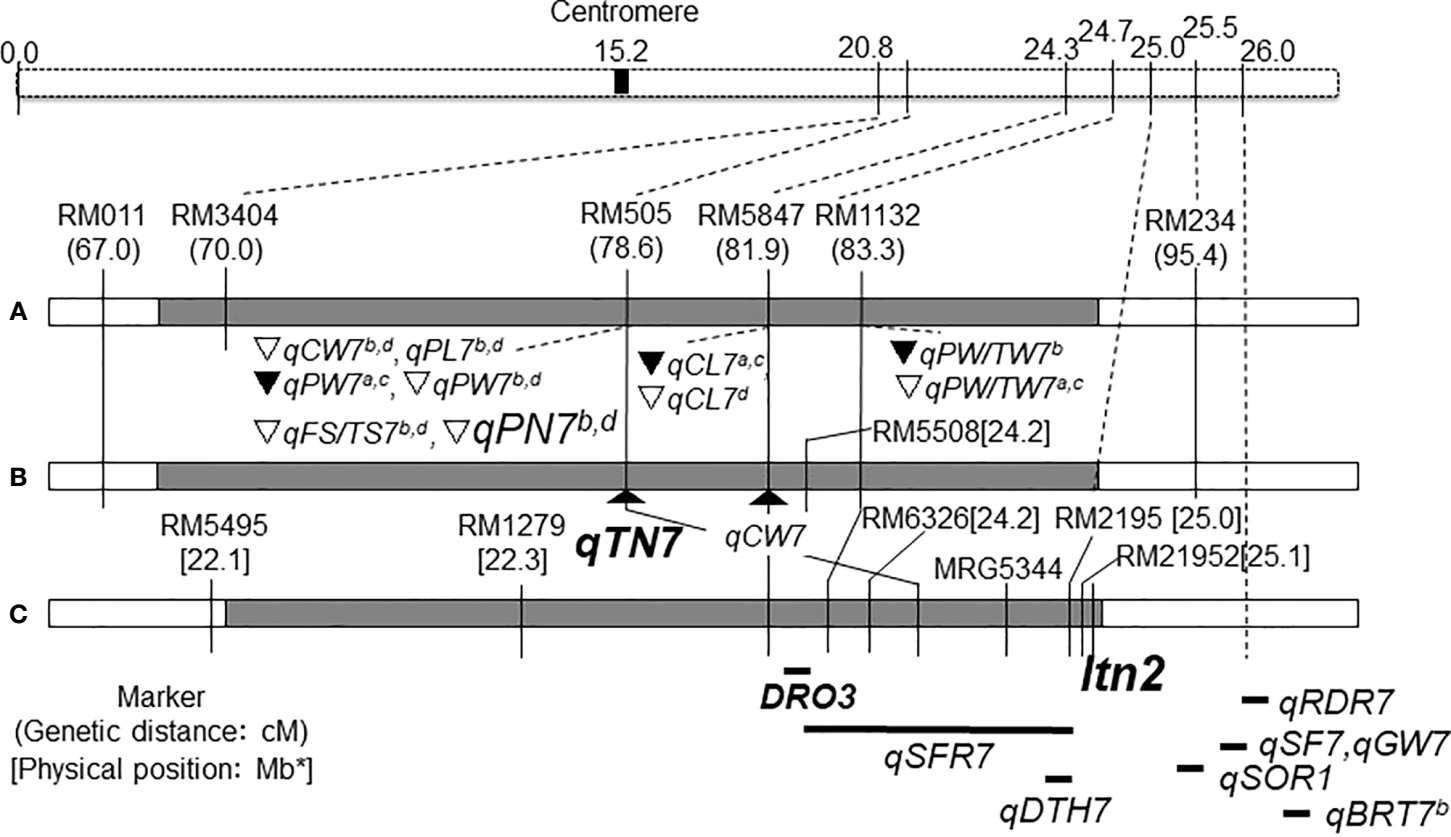
Choromosome location of low tiller number gene, *Itn2* and QTLs detected on chromosome 7. **(A)** Uddin et al. (2016a); **(B, C)** Uddin et al. (2016b); *DRO3*: Uga et al. (2015); *qSFR7*: Tomita and Fukuta (2019); qBRT7^a,b^: Li et al. (2011); *qRDR7*:Luo et al. (2015); *qSF7, qGW7*: Lafitte et al. (2004); *qSOR1*: Uga et al. (2012); *qDTH*: Monkada et al. (2001). ∇: YTH34 allele decreased value in upland.▼:YTH34 allele decreased value in irrigated lowland. CW, culm weight; PW, panicle weight; PW/TW, ratio of panicle weight to total plant weight; CL, culm length; PL, panicle length; PN, panicle number; FS/TS, ratio of fertile spikelet per panicle; BRT, Basal root thickness; RDR, Ratio of deep rooting; SOR, Soil surface rooting; SF, Spikelet fertility; GW, Grain weight; DTH, Days to heading. ^a^Irrigated lowland in 2011, ^b^Upland in 2011, ^c^Irrigated lowland in 2012, and ^d^Upland in 2012. *Physical position of the Chromosome 7 was redrawn from the genome database in Oryzabase (https://shigen.nig.ac.jp/rice/oryzabase/) using the Nipponbare reference genome. 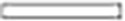 IR 64, 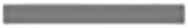 YTH 34, 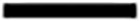 Flanked region of QTL.

International Rice Research Institute (IRRI) developed the New Plant Type (NPT) varieties characterizing as the low tiller numbers and a high productive tiller ratio by introducing the chromosome segments from the Tropical Japonica Group varieties, as one of the traits for genetic improvements to increase rice yield production (Khush, 2000). Fujita et al. (2009) reported 334 introgressions lines (INLs) with the genetic background of an Indica Group variety IR 64 and harboring several chromosome segments from NPT varieties which were bred by the crosses between Indica Group and Tropical Japonica Group upland varieties. In the other words, these chromosome segments in INLs were mainly originated from the Tropical Japonica Group upland varieties. Uddin et al. (2016a) found 18 QTLs for yield component traits including a QTL for low panicle numbers (as an indicator of tiller number), *qPN7*, on chromosome 7, by association analyses with 35 INLs harboring chromosome segments from the NPT variety, IR 65600-87-2-2-3 among them (**Figure 1**). IR 65600-87-2-2-3 harbors the chromosome segments of the Tropical Japonica Group upland rice variety, Ketan Lumbu. The other QTLs for culm length, panicle length, panicle weight, harvest index (panicle weight/total weight), and fertility rate were also found in the same region detected *qPN7* on chromosome 7. To confirm the detailed chromosome position of the QTLs detected, advanced genetic studies were conducted using two hybrid populations. A total of 88 F_3_ family lines derived from a cross between IR 64 and YTH34 which was one of 35 INLs harboring the segment on chromosome 7 from the NPT variety, IR 65600-87-2-2-3. The values of panicle numbers, spikelet numbers, and dry weight of whole plant in YTH34 were lower than those of IR 64 under the cultivation in irrigated lowland condition, and remarkable reductions of these values were observed under upland cultivation (**Table 2**) and an F_3_ population consisting of 72 plants, which was self-pollinated from an F_2_ plant, F_2_-JII-IV-10, harboring the heterozygote chromosome region for *qPN7* (Uddin et al., 2016b). These segregation analyses under upland conditions confirmed the single gene segregations. It was found a QTL for low tiller and designated as a recessive gene, *ltn2*, originated from the Tropical Japonica Group rice (**Figure 2**). Linkages were also found with some Simple Sequence Repeats (SSR) markers RM505, MRG5344, and RM21950. The genetic distances between RM505 and *ltn2* were 3.4 cM, and between RM21950 and *ltn2* they were 1.1 cM, and *ltn2* was mapped most closely with RM21950. Advanced QTL analysis also confirmed other QTLs for culm length, culm weight, panicle length, panicle weight, seed fertility, and harvest index in the same chromosome region. These QTLs decreased the traits’ values with the YTH34 allele. The INLs, YTH34, with IR 64 genetic background, is introduced only the genetic factor(s) for tiller/panicle numbers and those for the other traits locating the other chromosomes were not harbored. These results indicated that *ltn2* originated from a tropical Japonica Group upland rice variety, Ketan Lumbu, had a strong genetic effect for occurring of tiller and panicle with negative effects for the other traits. The ecotype of upland landrace rice is characterized such as traits; low panicle/tiller numbers, long and thick culm, and large panicle. These traits are controlled by several genetic factors which were distributed on the rice genome chromosomes in each, and these were accumulated in the genetic background of upland ecotype rice. The tiller/panicle numbers have been known as the most difficult trait to detect genetically, because of large environmental error (Sasahara, 1997). In the other words, these were easy to be influenced and changed by environmental conditions. To control the tiller/panicle numbers, the genetic factor(s) with strong effects may be needed under the serious and unfavorable environmental conditions of upland. The low tiller gene and the other QTLs detected on chromosome 7, which were originated from the Tropical Japonica Group variety, were estimated to play the important role for developing shoot architecture of upland rice with strong effects.

**Table 2:** Agronomic traits of IR 64 and YTH34 under irrigated lowland and upland.

Conditions	variety	Panicle No./plant	Culm length (cm)	Panicle length(cm)	Spikelet No./panicle	Seed fertility (%)	Panicle weight(g)	Dry weight (g)	Harvest index(%)	Days to heading
Irrigated lowland	IR 64	22.0	70.3	25.3	130.6	48.2	25.1	78.5	31.9	107.6
	YTH34	15.0 (68)	70.8 (100)	25.9 (102)	109.1 (83)	85.4 (177)	32.2 (128)	66.6 (84)	48.4 (151)	108.3 (100)
Upland	IR 64	47.0	51.3	25.9	138.0	63.4	51.9	140.0	38.0	140.0
	YTH34	17.0 (36)	39.9 (77)	22.8 (88)	102.0 (74)	66.6 (105)	14.6 (28)	48.0 (34)	30.0 (78)	151.0 (107)

Rice varieties were cultivated at Tropical Agricultural Research Front, JIRCAS, Ishigaki, Okinawa Japan, in 2013 (Date were refereed from Uddin et al., 2016b).

Mean values among 12 plants were calculated in each trait.

( ) = Relative value (%) in compared with that of IR 64.

## Genetic Factors for Root Architecture

One major QTL for soil-surface roots, *qSOR1*, was found using 124 recombinant inbred lines derived from a cross between an Indonesian lowland rice cultivar, Gemdjah Beton, with soil-surface roots and a Japanese lowland rice cultivar, Sasanishiki, was detected and localized on the long arm of chromosome 7 by Uga et al. (2012).

A QTL for deep root-growth-angle, *DRO1*, was found on chromosome 9, using 26 chromosome segment substitution lines (CSSLs) which were developed from the cross combination between an upland variety, Kinandang Patong (Tropical Japonica Group), and IR 64 as the recurrent parent (Uga et al., 2015). And another QTL, *DRO3*, was also found on the long arm of chromosome 7 using the progenies derived from a cross between IR 64 and a CSSL harboring *DRO1*. *DRO3* contributed to the deep root growth angle trait with the allele of Kinandang Patong. The nearest marker RM5508 for *DRO3* locates in the same chromosome regions of *ltn2* with the distance of 0.7 cM. Another QTLs for deep rooting, *qRDR7*, was also reported in the same region of chromosome 7 and the allele of an upland variety IRAT109 contributed to deep rooting in the genetic background of Zhenshan 97B which was a shallow rooting parent by Luo et al. (2015).

Tomita et al. (2017) tried to clarify the genetic variation for the root angle distributions using 97 accessions, and these were classified into two cluster groups, A and B. The accessions of cluster group A showed shallow rooting including the soil-surface root type, but and the numbers of accession were few. An INL, YTH16, by Fujita et al. (2009) harboring chromosome segments from a common NPT variety, IR65600-87-2-2-3, was also included. The accessions in cluster group B showed a wide variation from shallow to deep rooting types in both Indica and Japonica Groups, lowland and upland ecotypes, and landraces and improved types. There were no relationships between the root vertical angle and total root numbers among them. These findings indicated that the root angle distributions were not related with the differentiations between Japonica Groups and Indica Groups, among ecotypes for lowland and upland, and among degrees of genetic improvement, and the accessions with soil-surface root were rare and unique among natural variations. Tomita and Fukuta (2019) clarified that the soil surface-rooting in YTH16 was controlled by three QTLs on chromosomes 2, 5, and 7, and one QTL, *qSFR7*, on chromosome 7 had the biggest effect and played a main role. *qSFR7* was detected in the same region as *ltn2* by Uddin et al. (2016b). The QTL for soil surface-rooting on chromosome 7 is also one of the unique characters originated from the Tropical Japonica Group variety in rice. The genetic factor(s) for root architecture may also be relevant to upland adaptation together with other traits.

## Ecotype Differentiated Genes

With the basis of the rice genome sequence and annotation databases, NCBI: http://www.ncbi.nlm.nih.gov/gene/; TIGR: http://rice.plantbiology.msu.edu/, we found the physical locations of *MRG5433* on chromosome 7 and three putative and expressed genes close to the target region of the Japonica Group rice genome (cultivar: Nipponbare), such as Os07g0607500, Os07g0607700, and Os07g0607800, were found that were related to abiotic stress tolerance (Sottosanto et al., 2007; Zhang et al., 2008; Huang et al., 2009; Kera et al., 2012).

Using the entire genome resequencing data from a large panel of 84 upland and 82 irrigated lowland rice accessions, Lyu et al. (2014) reported some selective deviations in rice chromosomes, which were called as the ecotype differentiation genomic regions (EDRs). In the EDRs, several individual ecotype’s differentiated genes (EDGs) that are critical in upland adaptation or the phenotypical differentiation between the two ecotypes, lowland and upland, were also detected. A type-A response regulator (ARR) gene, *Os07g0449700*, on chromosome 7, is of special interest as it belongs to the ARR gene family induced by cytokinin, plays important roles in cytokinin signaling, and has impacts on root and shoot development. This ecotype differentiation of this ARR gene, *Os07g0449700*, may explain some differences of root and plant height between upland and irrigated rice varieties. Further reciprocal transgenic experiments between upland and irrigated rice will clarify the effects of *Os07g0449700* in the adaptation of upland rice.

Xia et al. (2014) reported seven selective loci that are ecotype preferable alleles expressed under drought stress by the analyses for 47 drought-responding expressed sequenced tags-simple sequence repeats (EST-SSRs) markers, using 377 rice landraces collected from China. These EST-SSRs markers are selected from the DNA transcribe regions and are closely related to the functional gene expressed under drought stress. Among the seven, two loci, *E3735* and *E4208*, were related to the differentiation between upland and lowland varieties, and these corresponded to *Os07g0260000* and *Os07g0546500*, which were detected on the rice chromosome 7, which may have an important effect on drought resistance or drought tolerance in rice.

## Conclusion

Crops adapted to different agro-ecosystems always promote the variation of agriculturally important genes (Xia et al., 2014). The knowledge and information regarding the genetic differentiation for eco-typical variations of traits and adaptations will greatly contribute to harnessing the genetic resources for the breeding and cultivation of rice under upland as well as unfavorable environments. We reviewed the involvement of a region on chromosome 7 in the genetic differentiations for rice ecotypes to lowland and upland ecosystems.

The *ltn2* region on chromosome 7 showed the unique reactions: low tiller, low dry matter production, short panicle length, and short culm lengths (Uddin et al., 2016b), and soil surface rooting (Tomita et al., 2017; Tomita and Fukuta, 2019). Usually, the upland landrace varieties are shown the unique characters: low tiller, heavy and big panicle, and short and early maturation, and these agricultural traits might contribute to avoid the risk of short water supply and to keep the stable yield production based on the minimalizing reproductive tillers under rainfed conditions. This unique shoot architecture of low tiller and panicle may be an important trait for adaptation to serious upland environmental conditions (Uddin et al., 2016b). YTH34 had a lower plant height, spikelet numbers, dry matter productions, and longer time to heading, and did not show good performances compared with that of IR 64 under the upland condition (**Table 2**). These results suggest that the genetic background of *ltn2* is efficient in the upland variety. This *ltn2* related to one of the genetic factors for the adaptation of rice cultivar to upland conditions might be very useful for the genetic improvement of rice cultivars under upland or unfavorable conditions with the other genetic factors.

YTH34 harboring *ltn*2 and the genetic information of the low tiller gene including linked SSR markers as well as the proposed candidate genes will be useful for the genetic modification of plant architecture in the rice variety, and for understanding the genetic mechanism of differentiation between lowland and upland ecotypes. The model plant type for adaptation to upland conditions will be able to be reconstructed by *ltn2*. The information of gene pyramiding based on *ltn2* will help us to understand the architecture of upland rice cultivars.

The ecotype differentiated genes such as *Os07g0449700* for root and shoot development (Lyu et al., 2014), EST-SSRs based selective loci E3735 and E4208 and their corresponding genes *Os07g0260000* and *Os07g0546500* for drought resistance (Xia et al., 2014), and other detected QTLs (Uga et al., 2015; Uddin et al., 2016a; Uddin et al., 2016b) on the rice chromosome 7 also play key roles in upland adaptation and result in the phenotypical differentiation. Clarification of the relationships among these candidate genes, *ltn2*, and QTL for the soil surface root on chromosome 7, will be investigated in future studies.

This review will not only help the geneticists to understand the underlying molecular basis of adaptive divergence, but also provide to breeder and agronomist valuable information for rice domestication and adaptation, especially on the upland/water stress and drought torelant in rice.

## Author Contributions

MNU prepared the manuscript basically, and YF made the plan for the conformance of the manuscript. The paper was prepared based on the PhD course study for MNU under the supervision of YF in University of Tsukuba.

## Conflict of Interest

The authors declare that the research was conducted in the absence of any commercial or financial relationships that could be construed as a potential conflict of interest.

## References

[B1] Bian J. He H. Shi H. Zhu C. Peng X. Li C. (2013). Dynamic QTL detection and analysis of tiller number before and after heading in Japonica rice. Aus. J. Crop Sci. 7 (8), 1189–1197.

[B2] FAO RMM (2018). Rice Market Monitor. Vol. XXI:. Available at: http://www.fao.org/3/I9243EN/i9243en.pdf (Accessed (accessed on February 20, 2020)).

[B3] Farooq M. Siddique K. H. M. Rehman H. U. Aziz T. Lee D. J. Wahid A. (2011). Rice direct seeding: Experiences, challenges and opportunities. Soil Till. Res. 111, 87–98. 10.1016/j.still.2010.10.008

[B4] Fujita D. Santos R. E. Ebron L. A. Telebanco-Yanoria M. J. Kato H. Kobayashi S. (2009). Development of introgression lines of an Indica-type rice variety, IR 64, for unique agronomic traits and detection of the responsible chromosomal regions. Field Crops Res. 14 (2), 244–254. 10.1016/j.fcr.2009.08.004

[B5] Fujita D. Ebron L. A. Araki E. Kato H. Khush S. G. Sheehy E. S. (2010). Fine mapping of a gene for low-tiller number, *Ltn*, in japonica rice (*Oryza sativa* L.) variety Aikawa 1. Theor. Appl. Genet. 120, 1233–1240. 10.1007/s00122-009-1251-7 20062964

[B6] Hasegawa Y. Yamamoto E. Ashikari M. Sazuka T. Miyao H. Hirochika H. (2005). Characterization and mapping of *tillering dwarf rice 2*, *tdr2* . Rice Genet. News. 22, 48.

[B7] Hittalmani S. Huang N. Courtois B. Venuprasad R. Shashidhar H. E. Zhuang J. Y. (2003). Identification of QTL for growth and grain yield-related traits in rice across nine locations of Asia. Theor. Appl. Genet. 107, 679–690. 10.1007/s00122-003-1269-1 12920521

[B8] Huang S. Taylor N. L. Narsai H. Eubel R. Whelan J. Millar A. H. (2009). Experimental analysis of the rice mitochondrial proteome, its biogenesis, and heterogeneity. Plant Physiol. 149, 719–734. 10.1104/pp.108.131300 19010998 PMC2633852

[B9] IFPRI (2009). The challenge of rice production. Washington, DC: International Food Policy Research Institute.

[B10] Ishikawa R. Maeda K. Harada T. Nuzeki M. Saito K. (1992). Genotypic variation of 17 isozyme genes among Japanese upland varieties in rice. Japan J. Breed. 42, 737–746. 10.1270/jsbbs1951.42.737

[B11] Ishikawa S. Maekawa M. Arite T. Onishi K. Takamura I. Kyozuka J. (2005). Suppression of tiller bud activity in tillering dwarf mutants of rice. Plant Cell Physiol. 46, 79–86. 10.1093/pcp/pci022 15659436

[B12] Jiang G. H. Guo L. B. Xue D. W. Zeng D. L. Zhang G. H. (2006). Genetic analysis and gene-mapping of two reduced-culm-number mutants in rice. J. Integ. Plant Biol. 48 (3), 341–347. 10.1111/j.1744-7909.2006.00224.x

[B13] Kera K. Takahashi S. Sutoh T. Koyama T. Nakayama T. (2012). Identification and characterization of cis, trans-mixed heptaprenyl diphosphate synthase from *Arabidopsis thaliana* . FEBS J. 279, 3813–3827. 10.1111/j.1742-4658.2012.08742.x 22883514

[B14] Khush G. S. (2000). “New plant type of rice for increasing the genetic yield potential,” in Rice Breeding and Genetics. Ed. Nanda J. S. (Enfield (NH): Science Publishers), 99–108.

[B15] Lafitte H. R. Price A. H. Courtois B. (2004). Yield response to water deficit in an upland rice mapping population: associations among traits and genetic markers. Theor. Appl. Genet. 109, 1237–1246. 10.1007/s00122-004-1731-8 15490102

[B16] Li X. Qian Q. Fu Z. Wang Y. Xiang G. Zeng D. (2003). Control of tillering in rice. Nature 422, 618–621. 10.1038/nature01518 12687001

[B17] Li Z. Mu P. Li C. Zhang H. Li Z. Gao Y. (2005). QTL mapping of root traits in a doubled haploid population from a cross between upland and lowland *Japonica* rice in three environments. Theor. Appl. Genet. 110, 1244–1252. 10.1007/s00122-005-1958-z 15765223

[B18] Li J. Wang D. Xie Y. Zhang H. Hu G. Li J. (2011). Development of upland rice introgression lines and identification of QTLs for basal root thickness under different water regimes. J. Genet. Genom. 38, 547–556. 10.1016/j.jgg.2011.08.005 22133686

[B19] Lin H. X. Qian H. R. Zhuang J. Y. Lu J. Min S. K. Xiong Z. M. (1996). RFLP mapping of QTLs for yield and related characters in rice (*Oryza sativa* L.). Theor. Appl. Genet. 92, 920–927. 10.1007/BF00224031 24166618

[B20] Liu G. F. Zhang Z. M. Zhu H. T. Zhao F. M. Ding X. H. Zeng R. Z. (2008). Detection of QTLs with additive effects and additive-by-environment interaction effects on panicle number in rice (*Oryza sativa* L.) by using single segment substituted lines. Theor. Appl. Genet. 116, 923–931. 10.1007/s00122-008-0724-4 18274724

[B21] Liu G. Zhu H. Liu S. Zeng R. Zhang Z. Li W. (2010). Unconditional and conditional QTL mapping for the developmental behavior of tiller number in rice (*Oryza sativa* L.). Genetica 138, 885–893. 10.1007/s10709-010-9471-y 20623365

[B22] Liu G. Zhu H. Zhang G. Li L. Ye G. (2012). Dynamic analysis of QTLs on tiller number in rice (*Oryza sativa* L.) with single segment substitution lines. Theor. Appl. Genet. 125 (1), 143–153. 10.1007/s00122-012-1822-x 22350178

[B23] Luo Q. Chen L. Mei H. Wei H. Feng F. Wang P. (2015). Quantitative trait locus mapping of deep rooting by linkage and association analysis in rice. J. Exp. Bot. 66 (15), 4749–4757. 10.1093/jxb/erv246 26022253 PMC4507776

[B24] Lyu J. Li B. He W. Zhang S. Guo Z. Zhang J. (2014). A genomic perspective on the important genetic mechanisms of upland adaptation of rice. BMC Plant Biol. 14, 160. 10.1186/1471-2229-14-160 24920279 PMC4074872

[B25] Miyamoto N. Goto Y. Matsui M. Ukai Y. Morita M. Nemoto K. (2004). Quantitative trait loci for phyllochorn and tillering in rice. Theor. Appl. Genet. 109, 700–706. 10.1007/s00122-004-1690-0 15221143

[B26] Monkada P. Martinez C. P. Borrero J. Chatel M. Gauch Guimaraes E. (2001). Quantitative trait loci for yield and yield components in an *Oryza sativa* × *Oryza rufipogon* BC_2_F_2_ population evaluated in an upland environment. Theor. Appl. Genet. 102, 41–52. 10.1007/s001220051616

[B27] Nagata K. Fukuta Y. Shimizu H. Yagi T. Terao T. (2002). Quantitative trait loci for sink size and ripening traits in rice (*Oryza sativa* L.). Breed. Sci. 52, 259–273. 10.1270/jsbbs.52.259

[B28] Purevdorj M. Kubo M. (2005). Future of rice production and consumption. J. Food Dist. Res. 35 (1), 128–142. 10.22004/ag.econ.27145

[B29] Raza A. Razzaq A. Mehmood S. S. Zou X. Zhang X. Lv Y. (2019). Impact of climate change on crops adaptation and strategies to tackle its outcome: A review. Plants 8:34. 10.3390/plants8020034 PMC6409995 30704089

[B30] Sasahara T. (1997). “Panicles,” in Science of the rice plant, vol. Vol. III . Eds. Matsuo T. Futsugara Y. Kikuchi F. Ymauchi H. (Tokyo, Japan: Genetics, Food and Agriculture Policy Research Center), 285–294.

[B31] Sottosanto J. B. Saranga Y. Blumwald E. (2007). Impact of *AtNHXI*, a vacuolar Na^+^/H^+^ antiporter, upon gene expression during short- and long-term salt stress in *Arabidopsis thaliana* . BMC Plant Biol. 7, 18. 10.1186/1471-2229-7-18 17411438 PMC1853094

[B32] Takeda T. Suwa Y. Suzuki M. Kitano H. Ueguchi-Tanaka M. Ashikari M. (2003). The *OsTB1* gene negatively regulates lateral branching in rice. Plant J. 33, 513–520. 10.1046/j.1365-313X.2003.01648.x 12581309

[B33] Tomita A. Fukuta Y. (2019). QTL analysis for soil surface roots originating from New Plant Type rice (*Oryza sativa* L.). Plant Breed. 138 (2), 154–162. 10.1111/pbr.12680

[B34] Tomita A. Sato T. Uga Y. Obara M. Fukuta Y. (2017). Genetic variation of root angle distribution in rice (*Oryza sativa* L.) seedlings. Breed. Sci. 67, 181–190. 10.1270/jsbbs.16185 28744171 PMC5515312

[B35] Uddin M. N. Obara M. Yanagihara S. Ishimaru T. Kobayashi N. Fukuta Y. (2016a). Genetic characterization of introgression lines with the genetic background of the Indica-type rice (*O. sativa* L.) cultivar IR64 under irrigated lowland and upland. Field Crops Res. 191, 168–175. 10.1016/j.fcr.2016.03.007

[B36] Uddin M. N. Tomita A. Obara M. Yanagihara S. Fukuta Y. (2016b). Identification of a low-tiller gene, *ltn2*, from a new plant type cultivar in rice (*O. sativa* L.). Breed. Sci. 66, 790–796. 10.1270/jsbbs.16143 28163595 PMC5282767

[B37] Uga Y. Hanzawa E. Nagai S. Sasaki K. Yano M. Sato T. (2012). Identification of *qSOR1*, a major rice QTL involved in soil-surface rooting in paddy fields. Theor. Appl. Genet. 124, 75–86. 10.1007/s00122-011-1688-3 21894467

[B38] Uga Y. Kitomi Y. Yamamoto E. Kanno N. Kawai S. Mizubayashi T. (2015). A QTL for root growth angle on rice chromosome 7 is involved in the genetic pathway of *DEEPER ROOTING 1* . Rice 8 (8), 8. 10.1186/s12284-015-0044-7 PMC4384719 25844113

[B39] Vergara B. S. Venkateswarlu B. Janoria M. Ahn J. K. Kim J. K. Visperas R. M. (1990). Rationale for a low-tillering rice plant type with high density grains. Philippines J. Crop Sci. 15 (1), 33–40.

[B40] Wu K.-S. Glaszmann J.-C. Khush D. S. (1988). Chromosomal location of ten isozyme loci in rice (*Oryza sativa* L.) through trisomic analysis. Biochem. Genet. 26, 303–320. 10.1007/BF00561468 3408480

[B41] Wu W. R. Li W.-M. Tang D.-Z. Lu H.-R. Worland A. J. (1998). Time-related mapping of quantitative trait loci underlying tiller number in rice. Genetics 151, 297–303. 10.1093/genetics/151.1.297 PMC1460454 9872968

[B42] Xia H. Zheng X. Chen L. Gao H. Yang H. Long P. (2014). Genetic differentiation revealed by selective loci of drought responding EST-SSRs between upland and lowland rice in China. PloS One 9 (10), e106352. 10.1371/journal.pone.0106352 25286109 PMC4186790

[B43] Xu P. Yang J. Ma Z. Yu D. Zhou J. Tao D. (2020). Identification and validation of aerobic adaptation QTLs in upland rice. Life 10:65. 10.3390/life10050065 PMC7281610 32423169

[B44] Yan J. Q. Zhu J. He C. X. Benmoussa M. Wu P. (1998). Quantitative trait loci analysis for the developmental behavior of tiller number in rice (*Oryza sativa* L.). Theor. Appl. Genet. 97, 267–274. 10.1007/s001220050895

[B45] Zhang H. Ohyama K. Boudet J. Chen Z. Yang J. Zhang M. (2008). Dolichol biosynthesis and its effects on the unfold protein response and abiotic stress resistance in Arabidopsis. Plant Cell 20, 1879–1898. 10.1105/tpc.108.061150 18612099 PMC2518237

[B46] Zheng H.-G. Babu R. C. Pathan M. S. Ali L. Huang N. Courtois B. (2000). Quantitative trait loci for root-penetration ability and root thickness in rice: comparison of genetic backgrounds. Genome 43, 53–61. 10.1139/g99-065 10701113

[B47] Zou J. Chen Z. Zhang S. Zhang W. Jiang G. Zhao X. (2005). Characterization and fine mapping of a mutant gene for high tillering and dwarf in rice (*Oryza sativa* L). Planta 222, 604–612. 10.1007/s00425-005-0007-0 16021500

